# Looking Tasks Online: Utilizing Webcams to Collect Video Data from Home

**DOI:** 10.3389/fpsyg.2017.01582

**Published:** 2017-09-12

**Authors:** Kilian Semmelmann, Astrid Hönekopp, Sarah Weigelt

**Affiliations:** Department of Developmental Neuropsychology, Institute of Psychology, Ruhr-Universität Bochum Bochum, Germany

**Keywords:** online experiment, video data acquisition, preferential looking, developmental psychology, methodology, infants

## Abstract

Online experimentation is emerging as a new methodology within classical data acquisition in psychology. It allows for easy, fast, broad, and cheap data conduction from the comfort of people’s homes. To add another method to the array of available tools, here we used recent developments in web technology to investigate the technical feasibility of online HyperText Markup Language-5/JavaScript-based video data recording. We employed a preferential looking task with children between 4 and 24 months. Parents and their children participated from home through a three-stage process: First, interested adults registered and took pictures through a webcam-based photo application. In the second step, we edited the pictures and integrated them into the design. Lastly, participants returned to the website and the video data acquisition took place through their webcam. In sum, we were able to create and employ the video recording application with participants as young as 4 months old. Quality-wise, no participant had to be removed due to the framerate or quality of videos and only 7% of data was excluded due to behavioral factors (lack of concentration). Results-wise, interrater reliability of rated looking side (left/right) showed a high agreement between raters, Fleiss’ Kappa, κ = 0.97, which can be translated to sufficient data quality for further analyses. With regard to on-/off-screen attention attribution, we found that children lost interest after about 10 s after trial onset using a static image presentation or 60 s total experimental time. Taken together, we were able to show that online video data recording is possible and viable for developmental psychology and beyond.

## Introduction

Over the last decade, psychological science has started to utilize the Internet as a new method of data acquisition. Scientists substitute pen and pencil questionnaires with online versions (e.g., Birnbaum, 2000; Gosling et al., 2000), record psychophysical data (e.g., Crump et al., 2013; Hilbig, 2015; Semmelmann and Weigelt, 2016), use twitter data to detect emotions in big data (Reips and Garaizar, 2011), or use web technology as an mediator of touch screen experiments (Frank et al., 2016; Semmelmann et al., 2016). The advantages of online experimentation are numerous: fast, low-cost, and parallel data acquisition that is independent of time and location is paired with the ability to access special populations, who might not be able to participate in in-lab studies (Birnbaum, 2004). These advantages broaden and speed up the experimental cycle of experiment creation, data acquisition, and analysis and allow scientists to reach conclusions to their questions in a faster, more efficient, and more unbiased way. Overall, since new technologies became available and more established, we see a shift from classical, in-lab-based data acquisition to online recordings.

To broaden the available methodology in online experimentation, this study explored the use of recently introduced web technology webcam access to conduct a classical developmental paradigm through the Internet. While it was possible to realize this approach before the new HyperText Markup Language (HTML) release (Jacobs, 2012), it heavily relied on downloadable plugins such as Flash or Java. After the introduction of HTML5, browsers natively support access to media devices, such as microphones and webcams, thereby offering better cross-browser support and an easy integration of this method. In short, processing data from webcams and microphones are not treated as a specialty anymore, but becomes a common approach in web technology.

Having access to a video recording device – the webcam of a computer – means that we can now deploy any paradigm of psychology that depends on having videos of the participant taken through online experimentation. Instead of relying on the strict, time-, and cost-intensive nature of in-lab measurements, we are able to present psychological paradigms online and acquire the data from participants, who are at home, in front of their own computer. Next to behavioral studies in adults, video material is especially relevant when considering research with infants (Franchak et al., 2011). While they might not yet physically or mentally be able to respond to a certain task as it is needed in classical psychophysics, their body language, head, and eye movements, which indicate attention attribution, can be used as metrics (von Hofsten, 1982; Colombo, 2001).

One of the most common tasks in this area is the preferential looking task (Fantz, 1965). In preferential looking, two stimuli are presented side-by-side and the experimenter usually records fixation durations to each stimulus: The stimulus receiving higher fixation durations is considered to be preferred by the infant participant. Since the initial studies, the variety of tasks has been increased and now covers preference of gender (Quinn et al., 2002), ethnicity (Kelly et al., 2008), familiarity (Matsuda et al., 2012), natural vs. unnatural faces (Valenza et al., 1996), color (Adams, 1987), and much more.

Eye gaze studies face several challenges, which might be overcome by online experimentation: Earlier studies [see Richardson and McCluskey (1983) for a review] suggest that about 25–75% of data recorded in looking tasks cannot be analyzed due to the young participants not being in a state of taking part in the experiment when they are present in the lab. They might fall asleep, cry, or simply not be interested in the display. While an online implementation could not solve the last issue, it could solve the first two. When parents are freely able to determine a good situation, in which they have time and their child is in an appropriate mood, they can use the moment and immediately participate in the study from home. The need to set an appointment with the researchers, travel to the institution, and hope that the child is in the right mood when arriving would then be superfluous. Still, even if the child would not be interested in the task, expenses of parents and researchers would be kept at a minimal level compared to in-lab data acquisition. Thus, we found a preferential looking task as particularly fitting for our endeavor as it is highly reliant on the participant on the one hand, but also a distinct differentiation between stimuli on the other hand. With regard to the latter part, we specifically chose a comparison between familiar and unfamiliar faces, which introduces the increased difficulty of obtaining the familiar images beforehand. Overall, researchers would save resources through online experimentation, while participants – both parents and infants alike – would have an increased level of comfort in their participation.

The main question we aspire to answer by employing a preferential looking task online is the technical feasibility. Is it possible to reliably record, save, and therefore analyze video recordings of babies through the Internet? Which factors need to be considered regarding Internet connections, transfer failures, and such? Furthermore, we aim at analyzing the data quality: Do we reach in-lab quality or are webcams not comparable with professional video equipment in this paradigm? Is there a difference between the active interest of children or do they still fall asleep and/or quit the experiment? And, while we do not want to present new findings about preferential looking in this first technical investigation, we are interested in whether researchers are reliably able to differentiate between the attention attribution of the young participants, so processible results through an online-based preferential looking task could be produced.

In sum, the present study is a first methodological investigation about the potential of conducting preferential looking tasks as they are commonly used in developmental psychology online. We try to provide a proof-of-principle through a technical realization of a classical experimental paradigm, carefully considering differences in data quality and seeing whether the data allow for analyzing task-specific effects. Thereby, we hope to answer the overarching question, whether an online approach to preferential looking tasks, or video-based online experimentation in a more general sense, is feasible.

## Materials and Methods

### General Procedure

**FIGURE 1:**
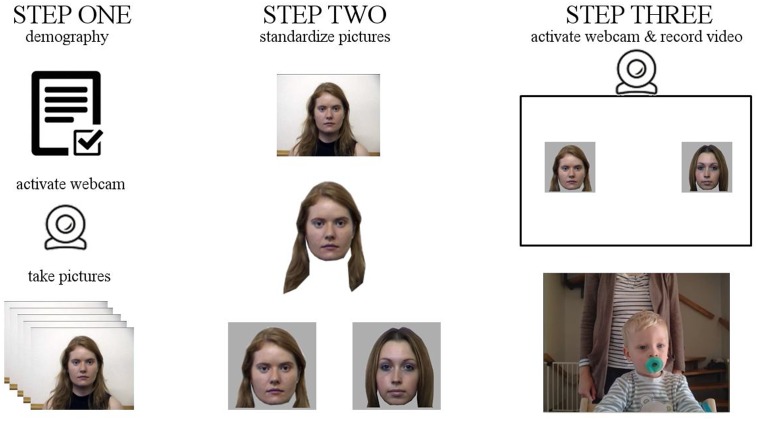
Three step process of the study. First, a registration took place, in which the participating parent was asked to take several photos through their webcam. In the second step, we processed the picture to fit a standardized appearance and matched it with a similar face from a stranger. Third, the experimental phase took place, in which the child was seated in front of the computer, the experimental trials were shown, and the webcam stream was recorded. Exemplary adult pictures are taken from PICS (Hancock, 2008), written consent for publication of the picture of the participating child was obtained from the mother.

Participation in the experiment consisted of three steps (**Figure 1**). First, potential participating parents visited a general website on which the background of the study, the researchers in charge, and the procedure to participate were explained. The experiment was approved by the local ethics committee and participating parents were only able to continue after agreeing to the consent information. If the consent information was accepted, a short questionnaire with regard to the demographics (gender of participating parent and child, age of child, potential developmental disorders) of the participants was presented. It was followed by instructions and an application taking several pictures of the participating parent that were used in the experimental part of the study. After submitting at least five images, the participating parent was informed that her/his data were going to be prepared for the experiment and she/he will be contacted via e-mail for participation.

In the second step, we examined the images and edited the pictures to fit the experimental procedure. Five images were selected and processed to only represent the face including hair, substituting the rest of the image with gray color. Those modified pictures were matched with other images that were either taken from the Psychological Image Collection at Stirling (PICS) database (Hancock, 2008)^1^ or other participating parents matching in gender, color of hair, and general appearance. The combinations of targets (picture of parent) and distractors (matched stranger images) were uploaded to the corresponding directory of the participating parent and an e-mail was sent to the participating parent through an automated system to inform them that the study was ready to continue.

The third step was the experimental task. Participating parents were able to revisit the website and were informed about the remaining procedure of the experiment. They were instructed (1) how to position the child in front of the computer, (2) to stand behind the chair out of the field of view of the child, (3) to not interact with the child during the experiment through speech, gestures, facial expressions, (4) to remove other potential distractions like mobile phones or music, and (5) reminded that they were free to quit the experiment at any time, for any reasons, and without any negative consequences. The last step of the setup instructed them to position the child centrally in front of the computer, to adjust the webcam accordingly and to start the paradigm, which started with a countdown of 10 s before the first trial appeared.

Each experimental trial was initiated by a centrally presented big schematic monkey face to attract the attention of the participating child. It was flashed for four times for 500 ms each, before the actual stimuli (faces of parent and stranger) were shown. Webcam stream recording started at the moment when the face stimuli appeared. The two faces were presented on the left and right side of the screen in a randomized order. They were shown for 15 s before disappearing and were followed by a gray screen for 1 s. In total, we conducted five trials in this manner. After one trial was finished, the data were uploaded simultaneously while the next trial was conducted to avoid unnecessary wait times after the experimental part.

After all five trials, the participating parent was presented with a progress bar for the remaining upload of files and a message that the experimental part was over and the child could be removed from the seating. Additionally, if not all uploads were finished yet, she/he was asked to wait for the uploads to finish before being able to continue. The last page of the experimental part was a thank you message, including the option to enter his/her e-mail address in case she/he wanted to take part in the raffle (due to low number of participants, every data contribution was awarded an Amazon voucher of 15€) and/or if she/he wanted to participate in further studies.

### Technology

The general parts of the website were realized through HTML5 and CSS, aided by JavaScript and the plugins jQuery 2.1.0, jQuery mobile 1.4.2, and jQuery UI 1.10.3. Form data were transferred through asynchronous JavaScript (AJAX) and saved through php 5.3.3 on an Apache 2.6.18 web server. The source code can be found at the Open Science Framework at https://osf.io/7eybq/.

Webcam access took place through the function getUserMedia. To allow processing the stream, participating parents had to accept a designated security request that followed initiating webcam access. If they declined, they were not able to continue and we were not able to access their data. In general, the stream was recorded and drawn onto a hidden canvas element, from which it was obtained and written to a file. To ease this process, we used the plugin RecordRTC (version of October 8th, 2015) and modified it to fit our purposes. Taking pictures of the participating parent and recording videos of the participating child were realized through the same technology, while in the former just a single frame was used and recorded without the plugin, and in the latter multiple frames were acquired through the method requestAnimationFrame, concatenated and sent as a singular video-file to the backend. To transmit data, we used the method $.ajax() of the jQuery package with a custom XMLHttpRequest. To account for unstable Internet connections, we used an incremental retry of data transmission that aborted after 10 failed attempts per file.

Depending on the browser, different approaches had to be used. Chrome provided video and audio streams in separate files, while Firefox allowed for a combined file. We did not support other browsers in this experiment, as these two browsers make up at least 70% of usage in 2015 (Buckler, 2016), which we consider sufficient for our participant recruitment efforts.

### Participation Rates

We distributed the URL to our experimental website through postings in message boards, Facebook, e-mails, and personal contact, and distributing flyers at local doctors’ practices, children shopping stores, kindergartens, and schools.

**FIGURE 2:**
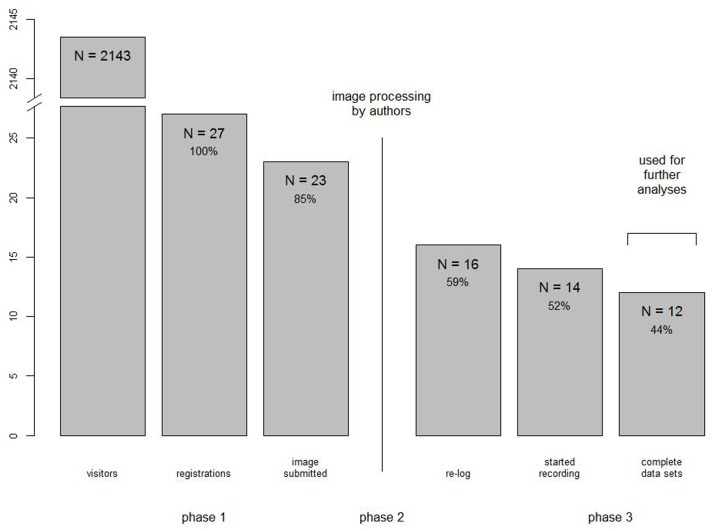
Participation rates in the study. Two of the 12 complete data sets had to be excluded due to age (*N* = 1) and behavioral factors (*N* = 1), therefore providing 10 data sets for rating analyses.

In total, we recorded 2143 unique visitors on our website through web log analysis (Total Exit Pages) over the course of 16 months (October 2015–January 2017; for a summary of participation rates, please see **Figure 2**). Due to our ethical agreement, we were not able to measure at which point a visitor quit our website before he/she accepted the consent form. Therefore, the number is just a rough estimate and includes non-human visitors (e.g., Google indexing bots), multiple visits, functional testing from the lab members, and possibly more. The first point of being able to save progress data was after participating parents accepted the consent form and entered their basic demographics. Here, we obtained 27 registrations, which equates to 1.26% of total visitors. Of the 27 registrations, four quit the study before pictures of the participating parent were taken. After the participating parents uploaded their pictures, we prepared the study, after which we sent each participating parent at least one automatic e-mail that they may proceed with the actual experiment now. Of the remaining 23 registrees, 7 did not re-log onto the website. Two more did re-log, read the instructions, but quit the experimental part at the moment they were asked to prepare the webcam for the preferential looking task. Therefore, in total, we recorded data from 14 participating children, which equals to 0.65% of total visitors and 52% of registrations.

Of the 14 data sets, in one case the upload of data failed, while in another only partial data were transmitted (two of five trials). As a consequence, we reinforced the iterative upload function by extending it to more tries with sequentially increasing time between each try of uploading the data, in the hopes of thereby accounting for temporary Internet connection loss. After the change, no more failed uploads were detected. The partial data sets were excluded from data quality analysis, thereby yielding 12 complete data sets.

### Data Quality Analysis

As a preliminary assessment of the possibility to analyze the results of a preferential looking paradigm, we performed a data quality check. Only data sets with complete video material were considered for this analysis (*N* = 12). To be able to compare those videos to in-lab quality, we assessed completeness of data, frame-rate, resolution, and brightness of the videos, and other potential distortive factors. If any of the video files would be rendered unusable due to the low quality, we would exclude them before performing the experimental analysis.

### Viewing Behavior Analysis

**FIGURE 3:**
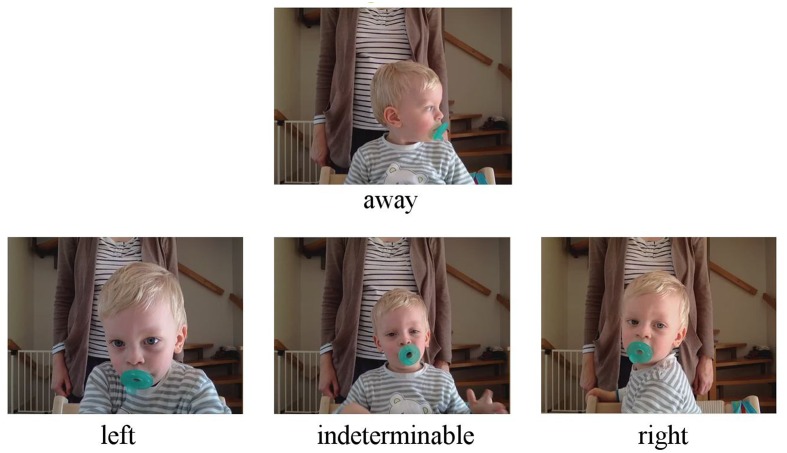
Video rating scale. Each video was split into 200 ms chunks and rated to either “left” or “right” side of the screen, an “indeterminable” position at the screen or “away” from the screen. Written consent for publication of the pictures of the participating child was obtained from the mother.

While the main focus of this paper is a methodological evaluation of the usability of webcam-based online recording of viewing behavior, we also analyzed data with regard to experimental effects. To quantify the attention attribution, we split each video into chunks of 200 ms and presented all chunks of all videos in a randomized order to three raters. The length of chunks was determined on the general assumption that saccades take at least 200 ms (Purves et al., 2001) forming the smallest expectable change in viewing behavior. Each chunk was independently rated by two of the authors and one additional colleague either as “left” (looking at the left side of the screen), “right” (looking at the right side of the screen), “indeterminable” (looking at the screen, but the rater was not able to determine a specific side), and “away” (not looking at the screen) in two sessions. For examples, please see **Figure 3**. To avoid potential experimenter’s biases, target position was not revealed until after the rating was finished, thereby creating a blind rating situation. Data were then analyzed with regard to how much time was spent looking on the screen (“How much data are usable?”) and with regard to effects of stimulus novelty (“Did the participating child spend more time looking at familiar or novel stimuli?”). Both questions were investigated on basis of trial data (cumulated over trials) and of total experimental time.

## Results

### Data Quality

As mentioned, the total number of started recordings was 14. Two of these recordings were not fully completed and therefore excluded, yielding an 86% completeness of data sets. Regarding image quality, we found all data sets fit for data analysis. Yet, one set had to be flipped horizontally, as the image was recorded upside-down. Another one was not well lit, thereby making analysis hard to perform, but still viable. All sets had a framerate of 3–30 fps (*M* = 17, *MD* = 15, *SD* = 10.82). All data were recorded in 640 × 480 px.

With regard to human factors, only one data set was influenced by a lack of concentration and participation (*N* = 1, age = 11 months). This data set was excluded from further analysis. One parent slightly intervened in the recording process through talking with the child, yet their data were included in the analysis because the child continued to pay attention to the stimuli. Another data set had to be excluded due to the age of the child (34 months) being way higher than our target group (2–24 months). Thus, overall, 10 complete data sets were usable for further analyses, while only one was excluded due to behavioral reasons (7%). The mean age of these children was 12.40 months (*SD* = 7.40, range 4–24 months), six were female (six female parents), four were male (three female and one male parents).

### Viewing Behavior

#### Interrater Reliability

Calculating Fleiss’ Kappa we found a moderate agreement (Landis and Koch, 1977) between raters, κ = 0.59 (57% agreement on all three raters), which rose to substantial agreement, κ = 0.73 (84%), when only differentiating between on-screen and off-screen viewing behavior and nearly perfect agreement, κ = 0.97 (98% agreement), when only considering cases, in which the rating of all raters was either “left” or “right” (preferential looking analyses). Thus, we can infer that all raters did agree whether participating children were looking at the screen and at which side, if they decided for one, but the agreement whether the gaze side was clearly identifiable or not was lower.

#### Screen Viewing

**FIGURE 4:**
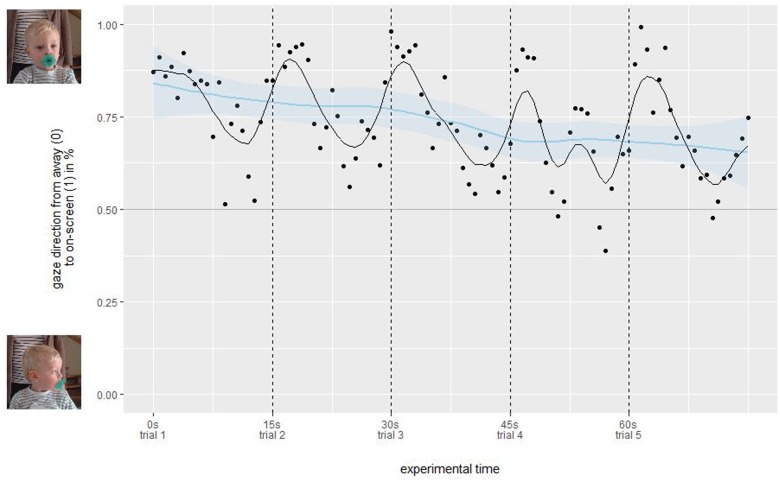
On-/off-screen differentiation over full experiment duration. Each dot denotes the average on-screen fixation in 100 ms bins in % from off-screen (0) to on-screen (1) over all participating children. The black line is a double five sample moving average, while the blue line denotes a LOESS fit over all data.

**FIGURE 5:**
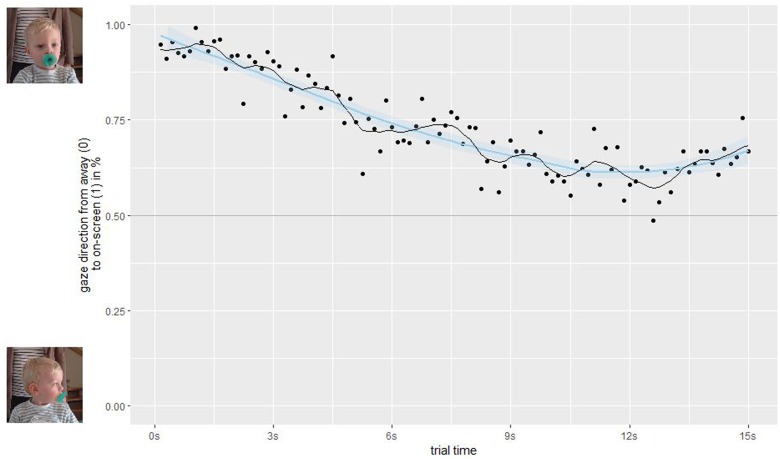
On/off differentiation averaged over trials. Each dot denotes the average on-screen fixation in 100 ms bins in % from off-screen (0) to on-screen (1) over all participating children and trials. The black line is a double five sample moving average, while the blue line denotes a LOESS fit over all data.

**FIGURE 6:**
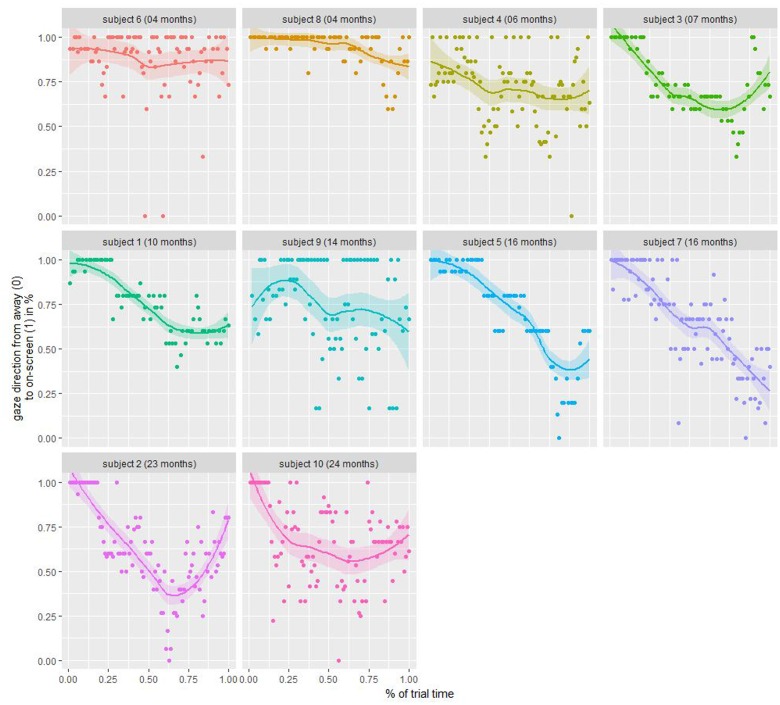
Individual on-/off-screen fixation. Each dot denotes the mean rating of three raters averaged over all trials whether the participant was looking on-screen (1) or away (0).

To analyze the attention retention, we calculated the duration a participating child was looking at the screen (ratings “left”, “right”, and “undefined”) against him/her looking away. **Figure 4** shows a clear loss of attention over the course of the whole experiment (binned into 100 ms bins) through a locally weighted scatterplot smoothing (LOESS) fitted curve (blue line), with an average on-screen time of 78% during the first trial and 71% during the last trial. A double five-sample moving average (black line) reveals additional oscillations during each trial, therefore, we averaged on-screen vs. off-screen time over all trials (**Figure 5**). Here, we find a sharp decrease from 92% during the first 3 s after trial start to 63% on-screen viewing for the last 3 s in each trial. Individually (**Figure 6**), we find that attention to the screen decreases over age. In sum, these investigations show that (1) the attention getter works well in attracting a child’s attention at the beginning of each trial, (2) after four trials (or 60 s) the child pays less attention to the screen, and (3) children between the age of 2 and 24 months lose interest for static on-screen images after about 10 s.

#### Preferential Looking Effects

**FIGURE 7:**
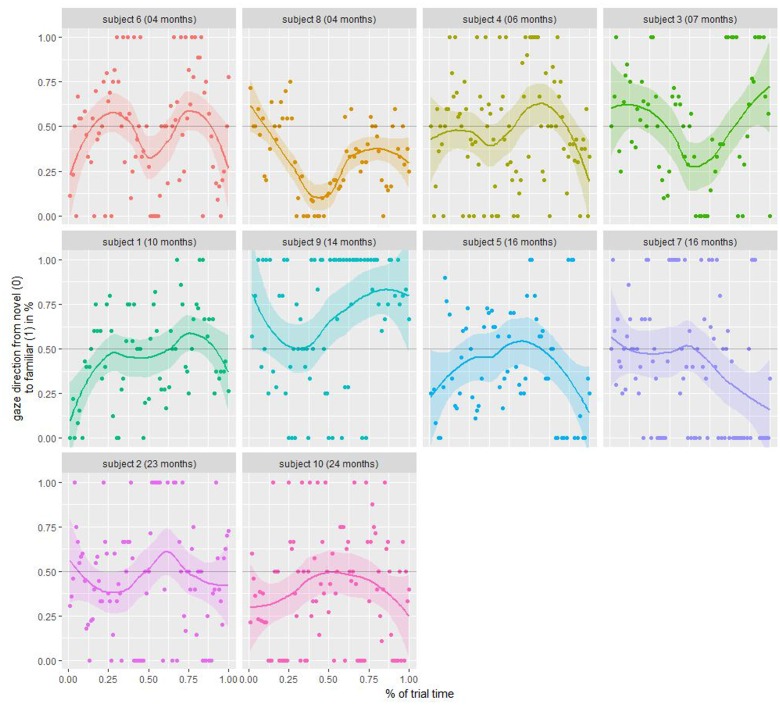
Individual familiar/novel fixation. Each dot denotes the mean rating of three raters averaged over all trials whether the participant was looking at the novel (0) or familiar (1) stimulus.

That preferential looking effects could be identified is already indicated by the high interrater reliability when examining the side preferences in the rating task. It shows that the video material is of a high enough quality to clearly identify which side is preferred by the participating children. Yet, to illustrate potential further preferential looking analyses, we present familiar/novel stimulus fixation graphs averaged over trials for individual subject in **Figure 7**. Here, only ratings categorized either as “left” or “right” were included and matched to the according underlying stimulus, thereby splitting the data into “novel” and “familiar” gazes. While on average we do have a slight preference for novel stimuli with 52, 55, 56, 56, and 52% in trials 1–5, respectively, we obviously cannot infer statistical conclusions due to the low sample size spread over a wide range of age. Still, we find oscillatory behavior that changes the preference about every 5 s (depending on age). Additionally, it seems to be individual, whether the child starts on a novel or familiar stimulus, as is the amplitude of preference. In sum, combined with the fact that raters reliably identified the side a participating child paid attention to, we can show that preferential looking effects can be analyzed, yet, statistical analyses were not conducted due to the low sample size.

## Discussion

This work used a preferential looking task to investigate the viability of webcam-based online experimental recording of children’s eye gaze between the ages of 2–24 months. We found the JavaScript/HTML5-based implementation working well with regards to technical feasibility and data quality, thereby producing processible data. Of those children that participated, attention to the task was held for up to 60 s of continuous viewing, while singular trials already showed a sharp decline in attention attribution after about 10 s. Also, that data can be reliably interpreted is shown by a very high interrater reliability analysis of on-screen vs. off-screen and side preference. Additional analysis of preferential looking behavior did not yield conclusive results due to the low sample size. Yet, we were able to coherently analyze data from all participating children between 4 and 24 months of age, thereby showing that the approach itself is viable over a large age range. In sum, we were able to establish a technical feasibility of the approach, recorded high quality data, and were able to analyze the data sets in the age range from 4 to 24 months.

### Technical Realization and Data Quality

Except for the data transmission issue mentioned above and already fixed during the data conduction phase where Internet connection loss led to loss of data, we did not find particular difficulties to setup the recording system and receiving the video files. A different HTML5 handling of different browser types is necessary, but is similar in implementation. The quality of data received on the other hand highly depends on the participant’s computer system. Lightning and positioning were not much of an issue in this study, yet, careful instructions need to be implemented to advise for an optimal recording environment. Instructions should cover seating position (distance, using a car seat or similar), avoidance of distractions (sound, persons, other browser tabs, moving the keyboard, and monitor out of reach), position of the parent (out of the field of view), and interactions (no talking, no pointing). Framerate on the other hand varied widely from 3 to 30 fps, which is a 10-fold increase in some systems, thus producing 10 times as many data samples on the high performing systems. Here, either it needs to be accounted for by excluding very low performing systems at analysis, or one could implement a pre-test that only allows participation, if the framerate is at a viable level. Still, even at the lowest framerate (3 fps), data were interpretable and could be used for analyses.

### Participation Rates

Despite various ways of recruiting for this study, the actual registration numbers were at a lower level than hoped. In our experience, participation rates in classical in-lab studies with children below 24 months of age are around 0.5% of total parents contacted (e.g., through mail based on birth registries), yet no scientific literature on this could be found. Whether the low conversion rate was due to privacy concerns or not having a webcam available cannot be determined due to the ethical agreement, which only allowed data recording after accepting the consent form. Nevertheless, we see four factors that could possibly be responsible, namely (1) the design of the study, (2) recruitment technique, (3) compensation, and (4) privacy concerns. The first factor is concerned with the three-step process we implemented to have an easy way of integrating parental stimuli into the experimental paradigm. We assume that due to the fact that parents had to visit and get engaged with our site twice (once to take photographs of themselves and then again to record the actual data), only intrinsically motivated visitors might have been willing to contribute, while casual visitors might have avoided the effort. Second, none of the recruitment techniques we used were particularly successful in attracting many contributions. We assume this is due to the lack of personal contact to researchers, who explain the intentions of the study and act as a contact person in case of questions, which entails privacy concerns, discussed later. The third factor might be compensation. Due to ethical considerations, we were not allowed to compensate every participant, but only to hold a raffle, to avoid money being the main motivator for parents to engage their child into research. Thus, one of the most effective tools in online research, the use of crowdscience platforms like Amazon Mechanical Turk, was not available to us. Lastly, privacy concerns are always a factor in online research. Allowing a website to take pictures of you and a video of your child might be discouraging to many parents, regardless of ethical approval and data privacy guidelines. We assume that those concerns, coupled with the three-step process and no guaranteed compensation led to rather low participation numbers.

### Further Studies

While we were able to confirm the main question of this study – the technical feasibility – the lack of power does not allow us to make inferential statements about the preferential looking results. This leads to further studies that could be improved in the following aspects. First, we would advise to avoid a complicated design that requires multiple engagements of participants, especially if they are new to the experimental process. A singular website with a coherent process of consent, registration, and experimental paradigm should lower the threshold of being repelled by the amount of effort. Secondly, we advise to either take into account or preemptively avoid very low performing systems, yet, this is dependent on the research question. Third, to increase participation rates, we think using the online system as an intermediate step by inviting known participants, which had personal contact to the researchers, to contribute would be helpful. This approach should lower preconceptions about the legitimacy of the research. Fourth, we found a clear decline of interest of the participating children after about 10 s per trial and 60 s experimental time. Therefore, we would advise to keep these numbers as low as possible to avoid sequential effects of attention attribution.

Obviously, all these recommendations and technical improvements are not only concerned with further preferential looking tasks, but also fit further paradigms that can be used through this approach (e.g., Habituation, Violation of Expectation). While the technical basis will stay the same (starting video recording through a webcam), additional factors will be introduced to different designs and requirements on stimulus presentation. Nevertheless, we think that with this work a cornerstone of the methodology has been set and can now be used by a variety of researchers in different fields.

### Summary

This work utilized web technology (HTML5 and JavaScript) to implement a preferential looking task for children between 2 and 24 months and record the according video material through the webcam of the participant. We found that the technological implementation and the resulting data quality are sufficient for the task, with a lower exclusion rate (7%) due to behavioral factors than classical in-lab studies (25–70% loss, Richardson and McCluskey, 1983). On the other hand, we did not find a clear preference for any of the stimuli types (novel, familiar); yet, this is in accordance with a current discussion about the interaction of habituation, individual preferences, age, and exposure time in such paradigms (Houston-Price and Nakai, 2004) and presumably amplified through our wide age range and low sample size. Still, we think this tool can be very helpful in conducting video-based data, especially in cases where parents already know the research institutions, longitudinal studies, specific design-related requirements (e.g., single trial experiments), to avoid inattentiveness or other behavioral factors, and to address replicability in developmental studies (e.g., Ebersole et al., 2016). Overall, while we have shown the technical viability of online preferential looking (or other video-based) data acquisition, specific strategies to recruit sufficient participants still need to be evaluated.

## Ethics Statement

This study was carried out in accordance with the recommendations of the Germany Association for Psychology (DGPs) with written informed consent from all subjects. All subjects gave written informed consent in accordance with the Declaration of Helsinki. The protocol was approved by the ethical approval board of the Institute of Psychology at Ruhr-Universität Bochum.

## Author Contributions

KS idea, programming, data analysis, and writing of paper; AH data collection, data analysis, and writing of paper; and SW idea, data analysis, and writing of paper.

## Conflict of Interest Statement

The authors declare that the research was conducted in the absence of any commercial or financial relationships that could be construed as a potential conflict of interest.

## Footnotes

1 http://pics.stir.ac.uk
